# Handgrip Strength and Healthspan: Impact of Sports During the Developmental Period on Handgrip Strength (Juntendo Fitness Plus Study)

**DOI:** 10.14789/jmj.JMJ23-0017-P

**Published:** 2023-07-24

**Authors:** TAKASHI ABE, YOSHIMITSU KOHMURA, KOYA SUZUKI, YUKI SOMEYA, JEREMY P. LOENNEKE, SHUICHI MACHIDA, HISASHI NAITO

**Affiliations:** 1 Institute of Health and Sports Science & Medicine, Juntendo University, Chiba, Japan; 1 Institute of Health and Sports Science & Medicine, Juntendo University, Chiba, Japan; 2 Graduate School of Health and Sports Science, Juntendo University, Chiba, Japan; 2 Graduate School of Health and Sports Science, Juntendo University, Chiba, Japan; 3 Department of Health, Exercise Science, and Recreation Management, Kevser Ermin Applied Physiology Laboratory, The University of Mississippi, MS, USA; 3 Department of Health, Exercise Science, and Recreation Management, Kevser Ermin Applied Physiology Laboratory, The University of Mississippi, MS, USA

**Keywords:** grip strength, physical activity, kendo athletes, predicting future health

## Abstract

Handgrip strength as a biomarker is being studied as a factor in predicting disease onset. However, the effect of improving handgrip strength through physical exercises, such as sports during the developmental period, on disease prevention has yet to be fully elucidated. The Juntendo Fitness Plus (J-Fit Plus) Study is a unique database of anthropometric and physical fitness measurements with over 50 years of accumulated data. It has the potential to explore the effects of sports on the association between handgrip strength and morbidity/mortality. We first outline previous studies on the impact of physical exercise interventions on handgrip strength, separated into adulthood and developmental period. We then introduced a unique effort to investigate the effects of sports using the J-Fit Plus Study database and describe the challenges of finally elucidating the impact of exercise on the association between handgrip strength and health status.

## Introduction

Handgrip strength is a well-known biomarker of the aging process and health. Many studies have reported the health benefits of higher handgrip strength in middle-aged and older adults over the quarter of a century^1-4)^. For example, recent large-scale follow-up studies have repeatedly revealed the inverse association between handgrip strength and various diseases, such as heart diseases^1)^, diabetes^2)^, cancer^3)^, and dementia^4)^. Although these analyses were adjusted for several covariates (age, education level, body mass index, alcohol, tobacco, medical history, etc.), the association still exists^1-4)^. In adolescents, low handgrip strength is also associated with poor cardiometabolic health outcomes^5)^ and premature death^6)^. Therefore, achieving and maintaining a high level of handgrip strength may be useful^7, 8)^. However, low handgrip strength is observed across the lifespan^9)^. If improving handgrip strength through physical activity and sports is beneficial for future health, it may be helpful as a strategy for children and adults with low handgrip strength. Therefore, this perspective aims to discuss the following three points: 1) the impact of an exercise training intervention on handgrip strength in adults, 2) the impact of physical activity and sports on handgrip strength in children and adolescents, and 3) future research directions to elucidate the association between handgrip strength and health status.

## Impact of exercise training intervention on handgrip strength in adults

Previous studies have examined the effects of nutrition alone, exercise alone, and combining the two on handgrip strength in middle-aged and older adults. For example, Bauer and colleagues^10)^ investigated the effect of dietary supplementation (i.e., vitamin D and leucine-enriched whey protein) on handgrip strength in 380 independent-living older adults. They reported that changes in handgrip strength did not differ from those in the control group after 13 weeks of the intervention. A meta- analysis has observed that nutrition alone has a small effect on handgrip strength (increased by 1.14 kg) compared to the control group^11)^.

On the other hand, Labott and colleagues^12)^ investigated the effects of exercise interventions on handgrip strength in healthy community-dwelling older adults of 60 years or older. They reported that the impact of exercise interventions, including aerobic and resistance training in different types or multi-dimensional training, was statistically significant, but the change in handgrip strength was small (standardized mean difference = 0.28). However, because this systematic review and meta-analysis include different modes of exercise training, it is necessary to consider the impact of resistance training alone, which might be particularly effective. Several systematic reviews and meta-analyses have recently reported the effects of resistance training on handgrip strength in older adults^13-15)^. Surprisingly, the impact of resistance training on handgrip strength in older adults was small (the standardized mean difference was less than 0.3). These results suggest that handgrip strength is unlikely to improve with regular resistance training during late adulthood^16)^. If this is true, it is thought that increasing handgrip strength as much as possible during the process of reaching young adulthood is effective in maintaining high handgrip strength during adulthood.

Incidentally, in the previous studies investigating the association between handgrip strength and morbidity, the morbidity was observed by dividing handgrip strength into three or four groups (e.g., low, mid, and high). In these studies, the differences in cutoff values or mean values between the low and high handgrip strength groups were approximately 10-15 kg^1, 3, 17, 18)^. These values may help inform on what change might be required to see an improvement in handgrip strength (at least one that might be associated with health benefits). However, that is a value that needs to be updated through longitudinal studies. More importantly, it will be important to identify the minimum level of strength required for health (if any).

## Impact of physical activity and sports on handgrip strength in children and adolescents

Resistance-type exercises for children and adolescents include lifting weights, bodyweight movements, and upper-body exercises performed during play from an early age. These physical activities are expected to contribute to developing upper-body and lower-body muscular strength, including handgrip strength for children and adolescents. A recent review^8)^ investigating the impact of physical activity and sports on handgrip strength in children and adolescents compared changes in handgrip strength in children engaged in extra physical activity and sports (i.e., intervention) to normal development (i.e., control). Types of interventions included family- and school-based physical activity and upper-body resistance training. The children and adolescents of the intervention groups increased the intensity and amount of physical activity at home or school, but no additional effects on handgrip strength were observed^8)^. Similarly, boys and girls ages 7-12 who underwent eight weeks of upper and lower body resistance training had no additional improvement in handgrip strength^8)^. This might suggest that using resistance training equipment for children is not enough of a stimulus for the agonist muscles of the finger flexors in the forearm and hand.

However, a recent study reported that handgrip strength might increase to a greater extent in those who participated in upper-body exercise (including gripping) during active play as children^19)^. There are sports where players hold equipment in their hands and those in which they do not. Therefore, we were interested to know what types of sports are more effective for improvement in handgrip strength during the developmental period. Fortunately, Juntendo University stores the results of handgrip strength measurements taken by students in school, and all first-year students undergo a handgrip strength test. This data only allows cross-sectional comparisons among sports events (i.e., an inability to know how much strength changed in response to the actual sport). However, first-year students usually have specialized in practicing one sport during their junior high and high school days.

## Analysis using Juntendo Fitness Plus (J-Fit Plus) Study data

Faculty of Health and Sports Science, Juntendo University has conducted anthropometric, physical fitness, and motor ability measurements, including handgrip strength, as part of the university curriculum, and the test results have been available since 1973. We used the J-Fit Plus Study data from 1973 to 2018 to investigate the effects of the type of sports practiced on handgrip strength in first-year sport university students. The faculty of Health and Sports Science used to be exclusively male students until 1991, and the initial analysis used data only for males^20)^. Privacy measures were maintained through Juntendo University, and all data were anonymized before analysis. We selected two types of sporting events with matching physiques (i.e., height and body mass), soccer (n=1127) targets the lower body, and kendo (n=297) and baseball (n=698) use the lower body simultaneously with upper body movement (including gripping). As a result, those in the lower body-only (soccer) sports had -3.78 (95% CI: -4.27, -3.29) kg lower handgrip strength than those in the lower + upper (kendo and baseball). Comparing each individual sport found that each sport was different from each other with kendo > baseball > soccer (between each sport, p<0.001)^20)^. The difference in handgrip strength between kendo and soccer was about 5 kg in the overall sample, but the difference between the two groups has widened since the beginning of data collection.

Next, we tried a similar comparison using data from female athletes. The J-Fit Plus Study database from 1996 to 2018 includes 2301 first-year female students, and we selected two sporting events, i.e., soccer (n=161) and kendo (n=53). The mean and standard deviation of age, height, and body mass in the soccer and kendo groups was 18.3 (0.9) years old, 159.7 (5.5) cm and 54.8 (5.8) kg, and 18.1 (0.3) years old, 159.2 (5.2) cm and 58.0 (7.7) kg, respectively. The handgrip strength of female kendo athletes was 34.0 (4.3) kg, which was significantly higher than that of female soccer athletes [27.9 (4.4) kg; difference of 6 (95% CI: 4.7, 7.4) kg]. The results did not appreciably change after height and body mass adjustment. Although these findings are cross-sectional, our female and male results suggest that performing sports activities with upper-body gripping movements may be able to augment strength during the developmental years. It is noted that the sports we selected in this study are natural activities that do not directly train handgrip strength.

## Future tasks

There are associations between handgrip strength and markers of health^1-6)^. However, the overarching question is whether increasing handgrip strength would lead to an improvement in health. Unfortunately, there is not enough evidence at the moment.

In adults, gripping exercise may improve handgrip strength, but this might not influence an individual's health status^8)^. At the same time, conventional (traditional) resistance exercise does not have a measurable influence^12-15)^. Therefore, handgrip strength acquired in early adulthood is unlikely to change, except for the influence of aging and illness. In addition, it is reported that low to moderate intensity (30%-50% of maximal voluntary contraction) isometric grip exercise training is associated with decreased blood pressure in men and women^21)^. However, this improvement in blood pressure is unlikely to be related to how strong (i.e., improving handgrip strength) someone can get. To the best of our knowledge, no studies have observed the impact of high-intensity grip training on risk factors for lifestyle-related diseases.

On the other hand, during the developmental period, sports such as kendo and baseball, which involve holding a tool with the hand, may positively affect the development of handgrip strength in children and adolescents^20)^. However, one of the critical future issues is whether the improvement in handgrip strength obtained by sports activity during the developmental period will change in later adulthood (especially the impact of stopping sports)^20)^. In addition, the J-Fit Plus Study is cross-sectional data and does not compare the changes in handgrip strength in each sport during development, even though athletes have been engaged in a single sport. Thus, it is necessary to confirm whether participating in these sports is able to augment handgrip strength over that of normal development using a longitudinal design. Although our cross-sectional work suggests this, it is still possible that those with better handgrip strength self-select sports that require a gripping component (i.e., kendo). Finally, it is possible to observe the morbidity of various diseases and mortality using the difference in handgrip strength according to sports events recognized in the J-Fit Plus Study data. As these studies develop, it may be possible to clarify what utility handgrip strength has as a biomarker.

## Funding

This study was supported by a grant from the Japan Society for the Promotion of Science Grants-in-Aid for Scientific Research (JSPS KAKENHI Grant Number JP22K11610) and the Institute of Health and Sports Science & Medicine, Juntendo University.

## Author contributions

TA, SM, JPL and HN have designed this study. YK, YS, KS, SM and HN have contributed to data collection. TA, YK and JPL have contributed to statistical analyses. TA wrote the first draft of the manuscript. All authors have contributed to the manuscript revision and read and approved the final version.

## Conflicts of interest statement

There are no conflicts of interest to declare.

## References

[1] Peralta M, Dias CM, Marques A, Henriques-Neto D, Sousa-Uva M: Longitudinal association between grip strength and the risk of heart diseases among European middle-aged and older adults. Exp Gerontol, 2023; 171: 112014. 10.1016/j.exger.2022.112014 36347359

[2] Boonpor J, Parra-Soto S, Petermann-Rocha F, et al: Associations between grip strength and incident type 2 diabetes: findings from the UK Biobank prospective cohort study. BMJ Open Diab Res Care, 2021; 9: e001865. 10.1136/bmjdrc-2020-001865 PMC8344322 34353878

[3] Parra-Soto S, Pell JP, Celis-Morales C, Ho FK: Absolute and relative grip strength as predictors of cancer: prospective cohort study of 445552 participants in UK Biobank. J Cachexia Sarcopenia Muscle, 2022; 13: 325-332. 10.1002/jcsm.12863 PMC8818619 34953058

[4] Esteban-Cornejo I, Ho FK, Petermann-Rocha F, et al: Handgrip strength and all-cause dementia incidence and mortality: findings from the UK Biobank prospective cohort study. J Cachexia Sarcopenia Muscle, 2022; 13: 1514-1525. 10.1002/jcsm.12857 PMC9178163 35445560

[5] Rioux BV, Kuwornu P, Sharma A, Tremblay MS, McGavock JM, Senechal M: Association between handgrip muscle strength and cardiometabolic z-score in children 6 to 19 years of age: Results from the Canadian Health Measures Survey. Metab Syndr Relat Disord, 2017; 15: 379-384. 10.1089/met.2016.0147 28759349

[6] Ortega FB, Silventoinen K, Tynelius P, Rasmussen F: Muscular strength in male adolescents and premature death: cohort study of one million participants. BMJ, 2012; 345: e7279. 10.1136/bmj.e7279 PMC3502746 23169869

[7] Buckner SL, Dankel SJ, Bell ZW, Abe T, Loenneke JP: The association of handgrip strength and mortality: What does it tell us and what can we do with it? Rejuvenation Res, 2019; 22: 230-234. 10.1089/rej.2018.2111 30200809

[8] Abe T, Thiebaud SR, Ozaki H, Yamasaki S, Loenneke JP: Children with low handgrip strength: A narrative review of possible exercise strategies to improve its development. Children (Basel), 2022; 9: 1616. 10.3390/children9111616 PMC9688465 36360344

[9] Ramirez-Velez R, Rincon-Pabon D, Correa-Bautista JE, Garcia-Hermoso A, Izquierdo M: Handgrip strength: Normative reference values in males and females aged 6-64 years old in a Colombia population. Clin Nutr ESPEN, 2021; 44: 379-386. 10.1016/j.clnesp.2021.05.009 34330493

[10] Bauer JM, Verlaan S, Bautmans I, et al: Effects of a vitamin D and leucine-enriched whey protein nutritional supplement on measures of sarcopenia in older adults, the PROVIDE study: a randomized, double-blind, placebo-controlled trial. J Am Med Dir Assoc, 2015; 16: 740-747. 10.1016/j.jamda.2015.05.021 26170041

[11] Wu PY, Huang KS, Chen KM, Chou CP, Tu YK: Exercise, Nutrition, and combined exercise and nutrition in older adults with sarcopenia: A systematic review and network meta-analysis. Maturitas, 2021; 145: 38-48. 10.1016/j.maturitas.2020.12.009 33541561

[12] Labott BK, Bucht H, Morat M, Morat T, Donath L: Effects of exercise training on handgrip strength in older adults: A meta-analytical review. Gerontology, 2019; 65: 686-698. 10.1159/000501203 31499496

[13] Manas A, Gomez-Redondo P, Valenzuela P, Morales JS, Lucia A, Ara I: Unsupervised home-based resistance training for community-dwelling alder adults: A systematic review and meta-analysis of randomized controlled trials. Ageing Res Rev, 2021; 69: 101368. 10.1016/j.arr.2021.101368 34022464

[14] Mende E, Moeinnia N, Schaller N, et al: Progressive machine-based resistance training for prevention and treatment of sarcopenia in the oldest old: A systematic review and meta-analysis. Exp Gerontol, 2022; 163: 111767. 10.1016/j.exger.2022.111767 35318104

[15] Grgic J, Garofolini A, Orazem J, Sabol F, Schoenfeld BJ, Pedisic Z: Effects of resistance training on muscle size and strength in very elderly adults: A systematic review and meta-analysis of randomized controlled trials. Sports Med, 2020; 50, 1983-1999. 10.1007/s40279-020-01331-7 32740889

[16] Buckner SL, Dankel SJ, Mouser JG, Mattocks KT, Jessee MB, Loenneke JP: Chasing the top quartile of cross-sectional data: Is it possible with resistance training? Med Hypotheses, 2017; 108: 63-68. 10.1016/j.mehy.2017.08.009 29055404

[17] Al Snih S, Markides KS, Ray L, Ostir GV, Goodwin JS: Handgrip strength and mortality in older Mexican Americans. J Am Geriatr Soc, 2002; 50: 1250-1256. 10.1046/j.1532-5415.2002.50312.x 12133020

[18] Metter EJ, Talbot LA, Schrager M, Conwit R: Skeletal muscle strength as a predictor of all-cause mortality in healthy men. J Gerontol A Biol Sci Med Sci, 2002; 57: B359-365. 10.1093/gerona/57.10.b359 12242311

[19] Macak D, Popovic B, Cadenas-Sanchez C, Madic DM, Trajkovic N: The effects of daily physical activity intervention on physical fitness in preschool children. J Sport Sci, 2022; 40: 146-155. 10.1080/02640414.2021.1978250 34533112

[20] Abe T, Kohmura Y, Suzuki K, et al: Athletes in sporting events with upper-body griping movements have greater handgrip strength than those in sporting events that prioritize the lower body. Am J Hum Biol, 2023; 35: e23891. 10.1002/ajhb.23891 36916960

[21] Carlson DJ, Dieberg G, Hess NC, Millar PJ, Smart NA: Isometric exercise training for blood pressure management: A systematic review and meta-analysis. Mayo Clin Proc, 2014; 89: 327-334. 10.1016/j.mayocp.2013.10.030 24582191

